# Sharing our knowledge in prevention and care of burn injury: Let's do better!

**DOI:** 10.4103/2321-3868.113326

**Published:** 2015-06-26

**Authors:** Jun Wu

**Affiliations:** Institute of Burn Research, State Key Laboratory of Trauma, Burns and Combined Injuries, Chongqing Key Laboratory for disease Proteomics, Southwest Hospital, Third Military Medical University, Chongqing, 400038 PR China

In the course of the millennia, fire, a natural as well as a man-made entity, has been promoting human cultural and technological development. However, just like other powerful forces, fire has two sides: one that is positive or helpful and another that is negative or harmful. Obviously, as human beings survive and prosper better with fire than without it, it follows that possible burn injuries will stay with human beings.

For a number of well-known reasons, burn injury remains a quite severe social and medical problem. “One World, One Standard” was the proposal as the aim of the 16^th^ Meeting of the International Society for Burn Injuries (ISBI) held in Edinburgh in 2012. That proposal outlines the ideal state of affairs, and indeed it is also our dream. On the contrary, all over the globe, patients with burn wounds are at present exposed to widely dissimilar conditions stemming from broadly different ethnic cultures, personal economic conditions, national health policies, and local medical traditions, which together end up determining profound dissimilarities in burn care. This inconsistent care is the reason for us launching an open-access journal, *Burns & Trauma*, as we wish to share on this platform several experiences, different data, various cases, and diverse ideas in a fast and easy way. Doctors, scientists, nurses, therapists, and social workers could improve the care of burn patients, promote the prevention of fire disasters, decrease postburn disabilities, and pursue a common dream: “No Burn Injury” or at least “One World, One Standard”.

**Table 1:** 

Access this article online	
**Quick Response Code**:	**Website**: www.burnstrauma.com
	**DOI**: 10.4103/2321-3868.113326

*Burns & Trauma* is an open access journal that aims to cover the latest and best achievements in all aspects of burn and trauma research. This new journal will provide a unique platform for the rapid publication of guidelines, original articles, review articles, case reports, letters, commentaries, and thematic issues on scientific research related to burn and trauma research. Themes include but are not limited to emergency medicine, wound healing, tissue engineering, intensive care, nutrition, bioengineering, shock, rehabilitation, immunology, infection, psychology, stem cells, organ damage, injury epidemiology, regeneration, and so on.

I wish to acknowledge the people who have enthusiastically contributed to the launch of *Burns & Trauma*: Antonino Gullo, Zhiyong Sheng, Zhengguo Wang, Xiaobing Fu, David Mackie, Basil A. Pruitt, Ronald G. Tompkins, Yongmin Yu, Ubaldo Armato, Xianchang Li, Xuetao Pei and the whole editorial team. Their suggestions, encouragement, and hard work will promote the growth of this forthcoming journal absolutely.

I sincerely hope *that Burns & Trauma* will act as a successful vehicle for sharing our knowledge and thereby significantly contributing to the prevention and the management of burn injuries.

